# Effects of biochar, waste water irrigation and fertilization on soil properties in West African urban agriculture

**DOI:** 10.1038/s41598-017-10718-y

**Published:** 2017-09-06

**Authors:** Volker Häring, Delphine Manka’abusi, Edmund K. Akoto-Danso, Steffen Werner, Kofi Atiah, Christoph Steiner, Désiré J. P. Lompo, Samuel Adiku, Andreas Buerkert, Bernd Marschner

**Affiliations:** 1 Institute of Geography, Department for Soil Science and Soil Ecology, Ruhr-Universität Bochum, Universitätsstr. 150, D-44780 Bochum, Germany; 2 Organic Plant Production and Agroecosystems Research in the Tropics and Subtropics, Universität Kassel, Steinstr. 19, D-37213 Witzenhausen, Germany; 3 Department of Soil Science, University of Ghana, Accra, Ghana

## Abstract

In large areas of sub-Saharan Africa crop production must cope with low soil fertility. To increase soil fertility, the application of biochar (charred biomass) has been suggested. In urban areas, untreated waste water is widely used for irrigation because it is a nutrient-rich year-round water source. Uncertainty exists regarding the interactions between soil properties, biochar, waste water and fertilization over time. The aims of this study were to determine these interactions in two typical sandy, soil organic carbon (SOC) and nutrient depleted soils under urban vegetable production in Tamale (Ghana) and Ouagadougou (Burkina Faso) over two years. The addition of biochar at 2 kg m^−2^ made from rice husks and corn cobs initially doubled SOC stocks but SOC losses of 35% occurred thereafter. Both biochar types had no effect on soil pH, phosphorous availability and effective cation exchange capacity (CEC) but rice husk biochar retained nitrogen (N). Irrigation with domestic waste water increased soil pH and exchangeable sodium over time. Inorganic fertilization alone acidified soils, increased available phosphorous and decreased base saturation. Organic fertilization increased SOC, N and CEC. The results from both locations demonstrate that the effects of biochar and waste water were less pronounced than reported elsewhere.

## Introduction

Crop production in sub-Sahara Africa must cope with low and erratic rainfall and low soil fertility caused by low soil organic carbon (SOC) contents, low cation exchange capacity (CEC), low water holding capacity and a labile soil structure^1,2^. In addition there is ample evidence that continued cultivation with low biomass returns leads to a rapid SOC decline^3^. High urbanization rates in West Africa will likely further increase the pressure on soil resources in the rural areas, where most staples come from, but also in peri-urban and urban areas^4^. In urban areas of Ghana and Burkina Faso, most available space is used for vegetable production to be sold on burgeoning nearby markets for self-sufficiency^5^. In this context, raw (unpurified) waste water is often used for irrigation. Globally, 50 Mio households rely on waste water irrigation for their livelihoods, despite an increasing awareness of the risks for farmer’s and consumer’s health caused by pathogens and occasionally heavy metals^6,7^. In Ghana and Burkina Faso, only a small proportion of waste water (7.9% in Ghana and 2.3% in Burkina Faso^8^) is, with varying success, purified in treatment plants. From a farmer’s perspective, waste water is not only a reliable year-round source for irrigation but also a source of nutrients to maintain soil fertility and crop yields. Compared to tap or well water, waste water has in most studies a higher pH and electric conductivity, higher concentrations of C, P, N, exchangeable Ca^2+^, Mg^2+^, K^+^ and Na^+^ ^9,10^.

It has often been claimed that the application of biochar is an effective measure to sustainably increase soil fertility and reducing the vulnerability against droughts in the long run^11^. Soil application of biochar is claimed to increase SOC, and to improve water holding capacity, nutrient retention, pH, microbial activity and soil structure^12,13^. In this context, it has to be emphasized that biochar can not only be produced from wood, which is an often scarce resource with multiple other higher value uses, but also from crop residues using simple kilns or gasifiers. If managed appropriately, it may then be a low cost soil amendment with a high adoption potential for local farmers. However, raw materials and production characteristics vary and lead to different biochar qualities^14^. Large uncertainty also exists regarding the interactions between waste water irrigation, biochar, fertilization and soil properties over time.

In view of the above, the aims of this field study were to determine the effects of biochar additions, fertilization as well as irrigation water quality and quantity on soil properties under typical urban vegetable production in Tamale (Northern Ghana) and Ouagadougou (Central Burkina Faso). To this end, the study reports results from five soil sampling campaigns carried out on two multi-factorial field experiments over a time period of two years.

## Results

### Soil organic C

**Figure 1:**
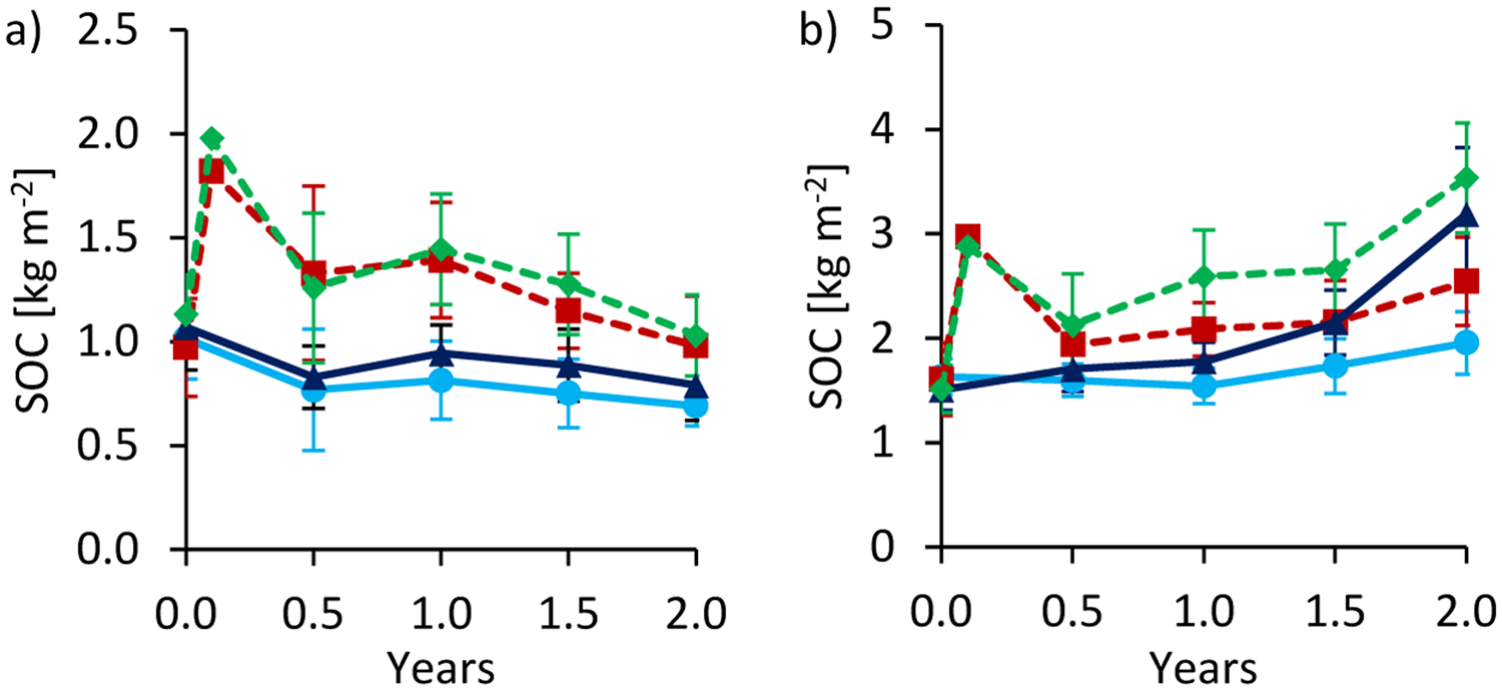
Changes of soil organic carbon stocks over time at 0–20 cm depth for Tamale (**a**) and Ouagadougou (**b**). Means were calculated irrespective of irrigation water quantity and quality levels because they had no significant effects on SOC stocks (means ± sd; n = 16). Values after biochar additions (between 0 and 0.5 years) are calculated and have no standard deviation.

Initially, biochar additions roughly doubled SOC stocks to 1.8 kg C m^−2^ in Tamale and to 3.0 kg C m^−2^ in Ouagadougou on a calculation basis (sum of initial SOC and biochar-C addition; Fig. 1). However, half a year after biochar addition, lower SOC stocks were measured at both sites, indicating a SOC loss in BC plots of 27 ± 13% in Tamale and of 35 ± 5% in Ouagadougou. In Tamale, SOC loss continued at a lower quantity over time and accounted for 46 ± 5% in BC plots after 2 years. Relative to the control, BC plots had only 38 ± 17% higher SOC stocks after two years, in Tamale. Further, in Tamale, control plots experienced a net SOC decline of 25 ± 13% (relative to the initial SOC stocks) after 0.5 years which increased to 32 ± 5% after two years.

In Ouagadougou, continuous manure fertilization in FP plots significantly increased SOC stocks by 0.76 kg m^−2^ per year (Fig. 1). Over two years, C input by manure was 2.4 kg m^−2^. Interestingly, measured SOC increase was only slightly lower than the amount of applied manure-C. After 0.5 years, 89% and after two years 82% of the applied manure C were found in the SOC stocks of FP plots. After 1.5 years, SOC stocks of FP plots exceeded those of BC plots. SOC stocks in control plots were lowest, but slightly increased, similar to BC plots, beyond 0.5 years in Ouagadougou. Relative to the control, BC plots had only 31 ± 15% higher SOC stocks while FP had 64 ± 19% higher SOC stocks, after two years in Ouagadougou.

Neither water quality nor water quantity significantly affected SOC stocks as main factors over time at both sites (Table 1). Mixed model analysis yielded significant effects for fertilization and the interaction of fertilization, water quality and time in Tamale. However, these effects were small and insignificant after adjustment of the mean comparison by the Tukey-Kramer method.

**Table 1:** Selection^1^ of mixed model type 3 tests for significances of main factor and interaction effects on SOC, total N, C/N ratio and pH over time in Tamale and Ouagadougou. Effects which were significant at p ≤ 0.05 are printed bold.

	**Tamale**				**Ouagadougou**			
	**SOC**	**Total N**	**CN**	**pH**	**SOC**	**Total N**	**CN**	**pH**
	log kg m^−2^	log g m^−2^	log		log kg m^−2^	log g m^−2^	log	
Block	0.341	0.768	**0**.**021**	0.226	**<0**.**0001**	**0**.**004**	0.978	**0**.**001**
Fert	**0**.**002**	**0**.**001**	0.657	**<0**.**0001**	**<0**.**0001**	**<0**.**0001**	**<0**.**0001**	**<0**.**0001**
Biochar	**<0**.**0001**	** < 0**.**000**	**<0**.**0001**	0.366	**<0**.**0001**	0.945	**<0**.**0001**	0.765
Quality	0.244	0.268	0.641	0.098	0.096	0.444	0.549	0.393
Quantity	0.761	0.812	0.858	0.286	0.333	0.661	0.686	**0**.**049**
Time	**<0**.**0001**	**<0**.**0001**	**<0**.**0001**	**<0**.**0001**	**<0**.**0001**	**<0**.**0001**	**<0**.**0001**	**<0**.**0001**
Quality*Quantity	0.992	0.792	0.733	0.319	0.827	0.962	0.757	0.350
Fert*Quality	0.972	0.706	0.254	0.664	0.701	0.823	0.899	0.221
Fert*Quantity	0.750	0.985	0.797	0.279	0.089	0.344	0.519	0.774
Fert*Quality*Quantity	0.845	0.426	0.143	0.276	0.802	0.231	**0**.**012**	0.077
Biochar* Quality	0.676	0.570	0.929	**0**.**027**	0.565	0.446	0.588	0.360
Biochar *Quantity	0.389	0.520	0.577	0.921	0.807	0.649	0.224	0.416
Biochar*Quality*Quantity	0.305	0.198	0.683	0.383	0.860	0.496	0.130	0.538
Fert*Biochar	0.075	**0**.**035**	0.855	0.274	0.670	0.080	** <0**.**000**	**0**.**011**
Fert* Biochar*Quality	0.079	0.196	0.054	0.079	0.355	0.482	0.969	0.761
Fert* Biochar*Quantity	0.566	0.553	0.795	0.799	0.096	0.641	0.123	0.562
Fert* Biochar*Quality*Quantity	0.231	0.494	0.076	0.894	0.416	0.147	0.095	0.564

Fert: Fertilization.

^1^The mixed model was run with all possible factors and factor interactions. Not presented are interactions with time.

**Figure 2:**
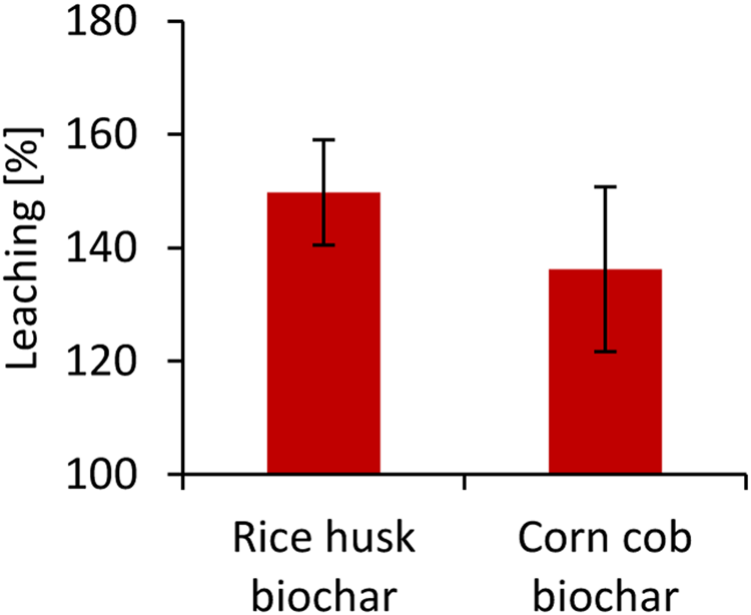
Cumulative DOC leaching of rice husk and corn cob biochar relative to control (100%) measured over 12 days with continuous drip wise waste water irrigation (J. Werner, unpublished).

To further clarify SOC losses, DOC leaching and CO_2_ evolution were studied in laboratory experiments. A column experiment with continuous drip wise waste water irrigation over 12 days demonstrated a 50% and 36% higher cumulative DOC leaching (particle size <0.45 µm) under soils amended with rice husk biochar and corn cob biochar, respectively (at 5 kg m^−2^ biochar), than in the control soil from the Tamale site (Fig. 2, J. Werner, unpublished).

**Figure 3:**
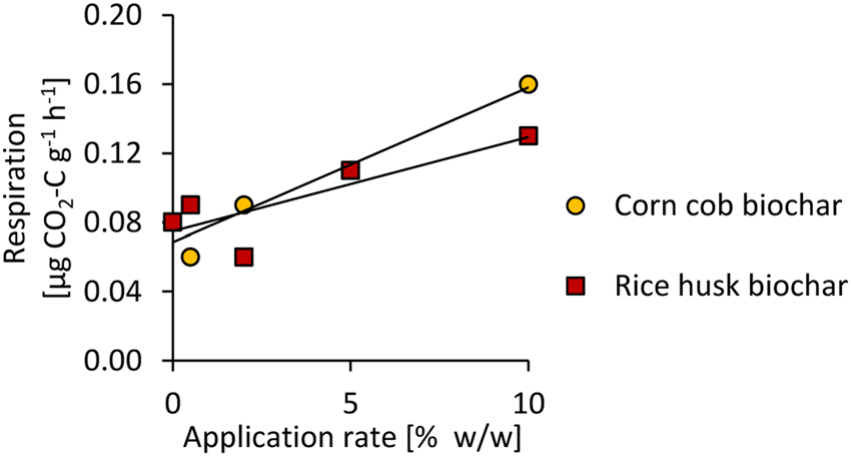
CO_2_ evolution measured over 60 days in a laboratory incubation of soil amended with rice husk and corn cob biochar at various application rates (A. Neuser, unpublished).

CO_2_ evolution measured over 60 days in a lab incubation of soil from the experimental site in Tamale amended with rice husk and corn cob biochar demonstrated increasing CO_2_ emissions with increasing biochar addition rates (Fig. 3, A. Neuser, unpublished).

In Tamale, SOC stocks at 20–40 cm depth remained similar over time whereas in Ouagadougou, manure fertilization increased SOC stocks also in 20–40 cm by 13%, relative to the control (data not shown). No change was observed for biochar, water quality and quantity.

### Total N

**Figure 4:**
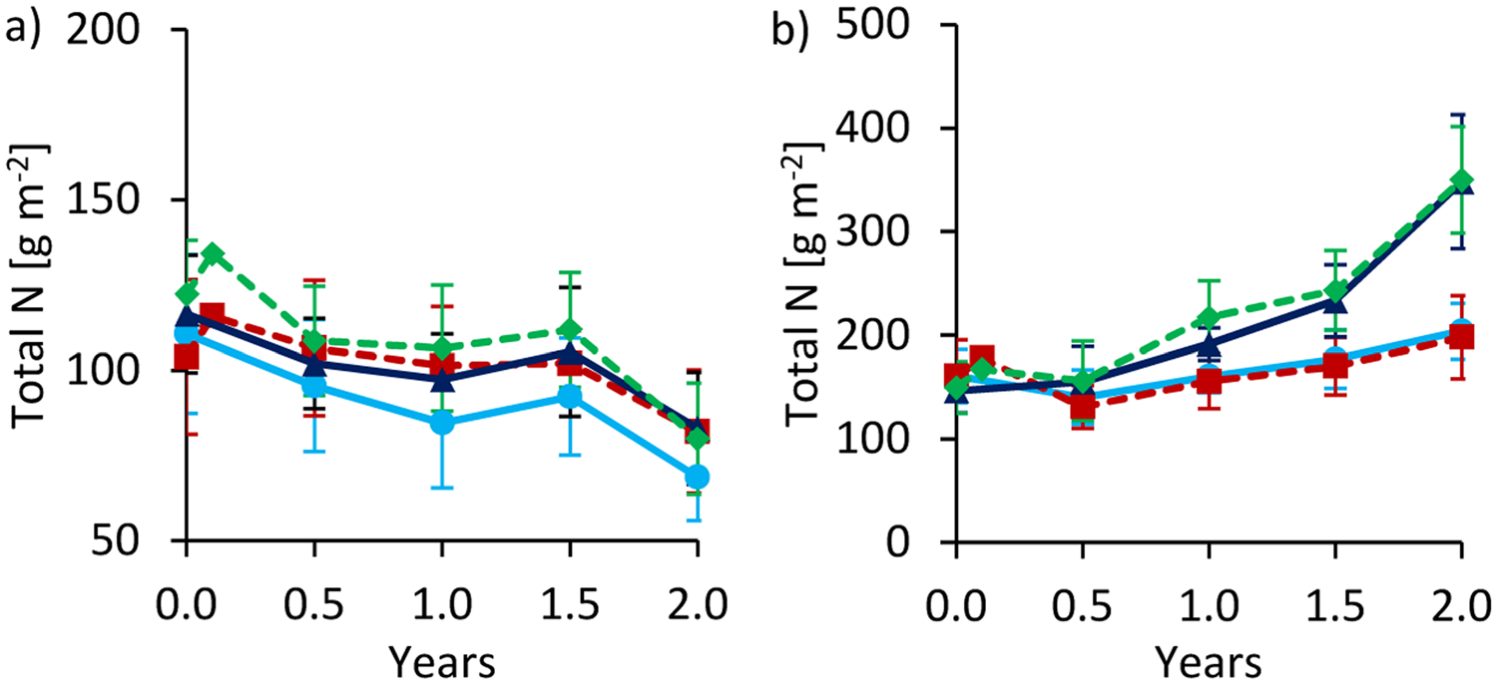
Changes of total N stocks over time at 0–20 cm depth for Tamale (**a**) and Ouagadougou (**b**). Means were calculated irrespective of irrigation water quantity and quality levels because they had no significant effects on N stocks (means ± sd; n = 16). Values after biochar additions (between 0 and 0.5 years) are calculated and have no standard deviation.

In Tamale, total N stocks were significantly affected by biochar (Table 1). Over time, all plots in Tamale experienced a net N decline which was strongest in control plots with 38 ± 6% of initial N stocks after two years (Fig. 4). The largest biochar effect was observed under clean water without fertilization with 18 ± 11% higher N stocks for BC than for control plots (average from 0.5 to 2.0 years). Biochar addition increased initial N stocks by 12%. In Tamale, inorganic N input from waste water (61 g N m^−2^) was similar to N input from inorganic fertilizer (59 g m^−2^) over two years. In line with SOC, mixed model analyses yielded significant effects of fertilization and the interaction of fertilization, water quality and time on N stocks, however, these effects were small and insignificant according to the Tukey-Kramer adjusted mean comparisons.

In Ouagadougou, N stocks significantly increased with organic fertilization at a rate of 96.4 g m^−2^ per year, thus, more than doubled (2.4 fold increase) within two years. Over two years N input by manure was 186 g m^−2^. In contrast to C, measured N increase over time and manure-N additions were of similar magnitude. No significant effects on N stocks were found for biochar in Ouagadougou, despite biochar N additions of 11%, relative to initial N stocks. In control and BC plots a slight increase of N stocks was observed over time irrespective of irrigation. No significant main effects on N stocks by water quality and quantity were found at both sites. In Ouagadougou, inorganic N fertilization (105 g m^−2^) was higher and waste water N input (12 g m^−2^) was lower than in Tamale over two years but the sum of inorganic N inputs was similar for both sites.

### C/N ratio

**Figure 5:**
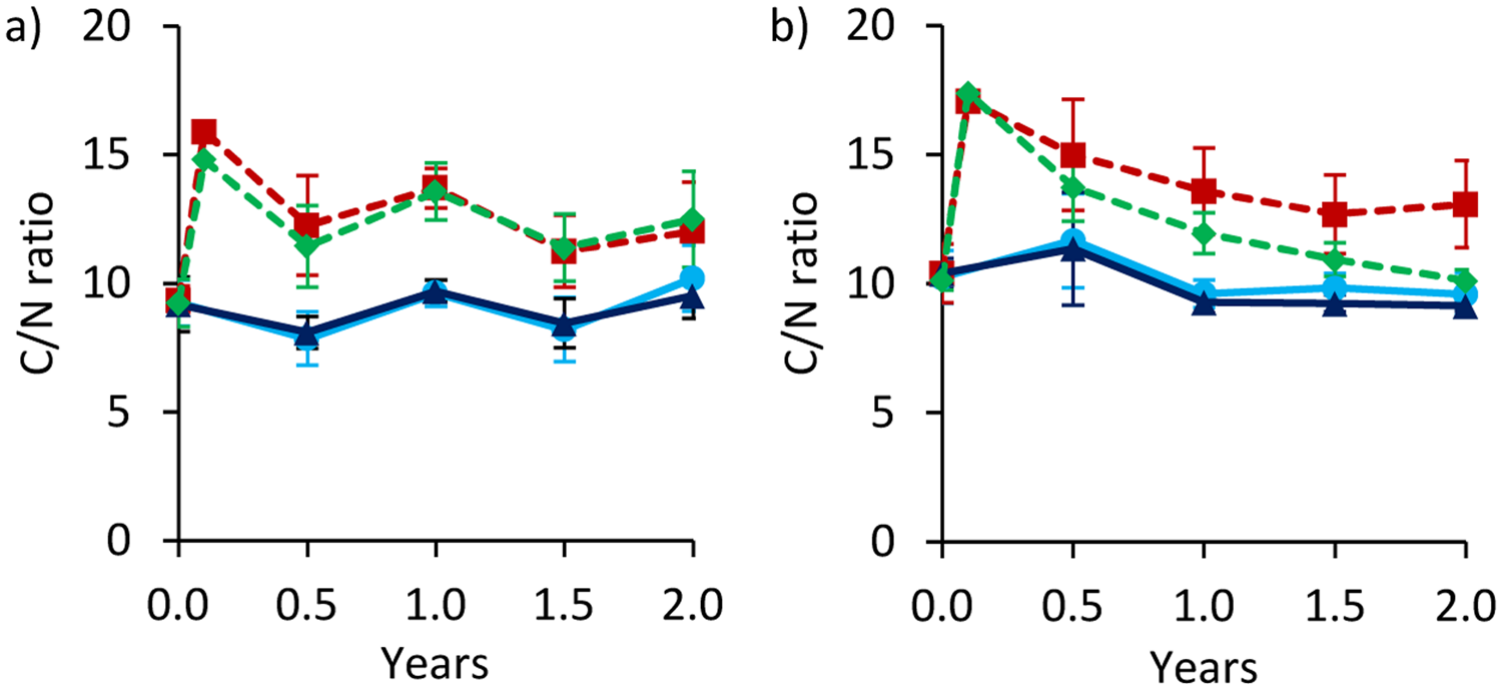
C/N ratio changes over time at 0–20 cm depth for Tamale (**a**) and Ouagadougou (**b**). Means were calculated irrespective of irrigation water quantity and quality levels because they had no significant effects on C/N ratios (means ± sd; n = 16). Values after biochar additions (between 0 and 0.5 years) are calculated and have no standard deviation.

At both sites, C/N ratios were significantly affected by biochar and in addition by fertilization in Ouagadougou (Fig. 5; Table 1). Biochar addition increased C/N ratios to 15.3 in Tamale and to 17.2 in Ouagadougou (on a calculation basis). After addition, C/N ratios of BC plots decreased rapidly during 0.5 years to 11.8 in Tamale and to 14.4 in Ouagadougou. In contrast to BC, C/N ratios of FP+BC decreased over time to ratios which were similar to control plots after two years in Ouagadougou. The C/N ratios of the non-biochar plots (FP and control) remained largely unchanged over time at both sites. No significant effects of water quality and quantity were observed.

### pH

**Figure 6:**
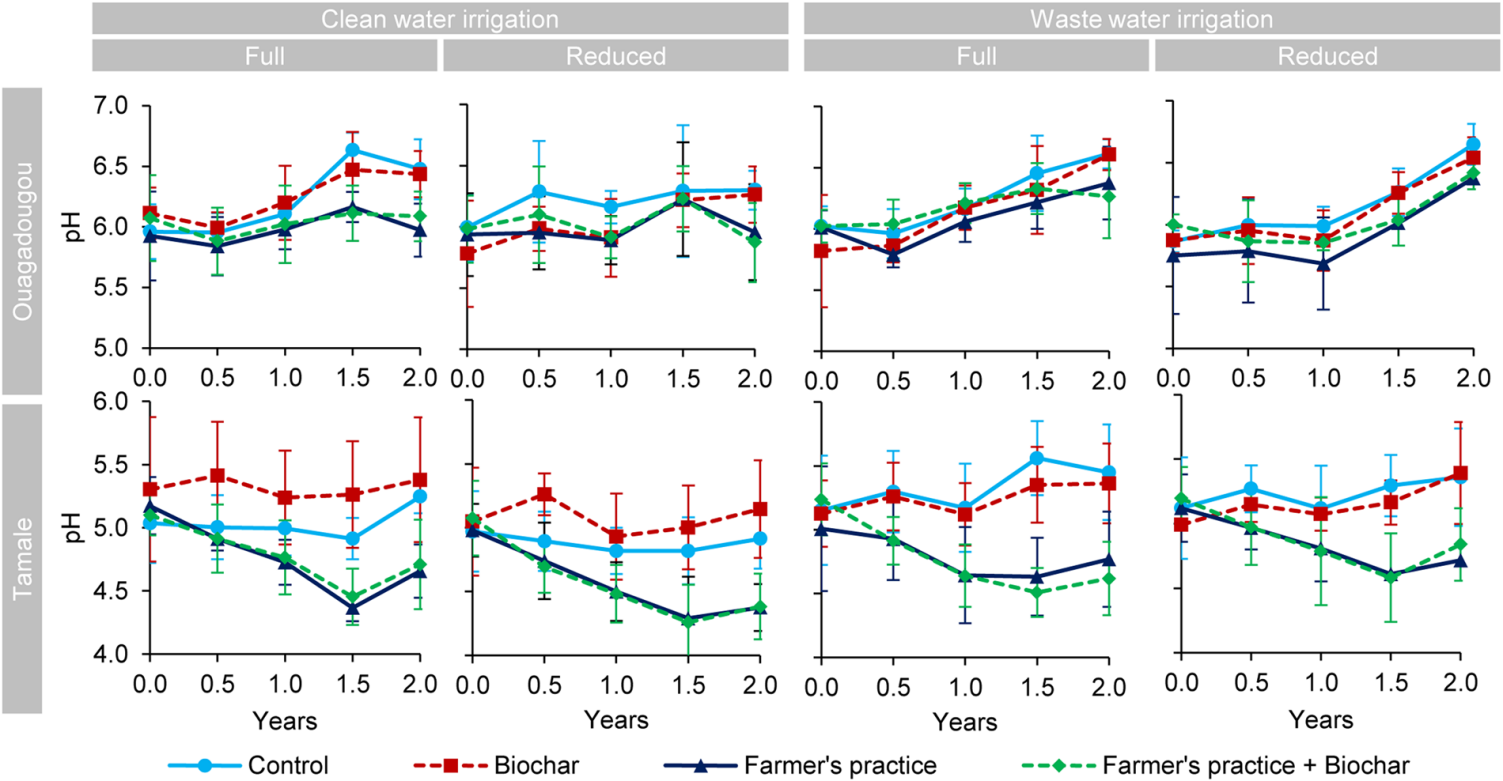
Soil pH changes over time at 0–20 cm depth grouped by irrigation quality and quantity levels for Tamale and Ouagadougou (means ± sd; n = 4).

Biochar had no significant effects on pH at both sites. In Tamale, pH strongly declined (by 0.65 ± 0.15 units) on all fertilized plots (FP and FP+BC) until 1.5 years after which pH leveled at 4.55 ± 0.18 (Fig. 6, Table 1). Further, pH increased significantly over time in plots under waste water irrigation, most remarkably in control plots which had a pH of 5.32 ± 0.14 under waste water and a pH of 4.95 ± 0.14 under clean water in Tamale beyond 0.5 years. In line, pH decline of the fertilized plots was slightly smaller under waste water (0.56 ± 0.15) than under clean water (0.74 ± 0.08).

In Ouagadougou, fertilization, the interaction of water quality and time (data not shown) and (to a small degree) water quantity affected pH significantly over time (Table 1). Waste water led to a higher and a more consistent increase of pH than clean water over time, irrespective of fertilization and biochar levels. This increase was most pronounced after two years with a pH of 6.48 ± 0.14 under waste water and 6.17 ± 0.23 under clean water irrigation. Fertilized plots (FP and FP+BC) had slightly lower pH values (6.14) than unfertilized plots (6.55), most pronounced after 1.5 years under full clean water irrigation. Water quantity levels caused a minor significant effect on pH for some treatments, but these effects were isolated at the sampling after the first year.

### Available P

**Figure 7:**
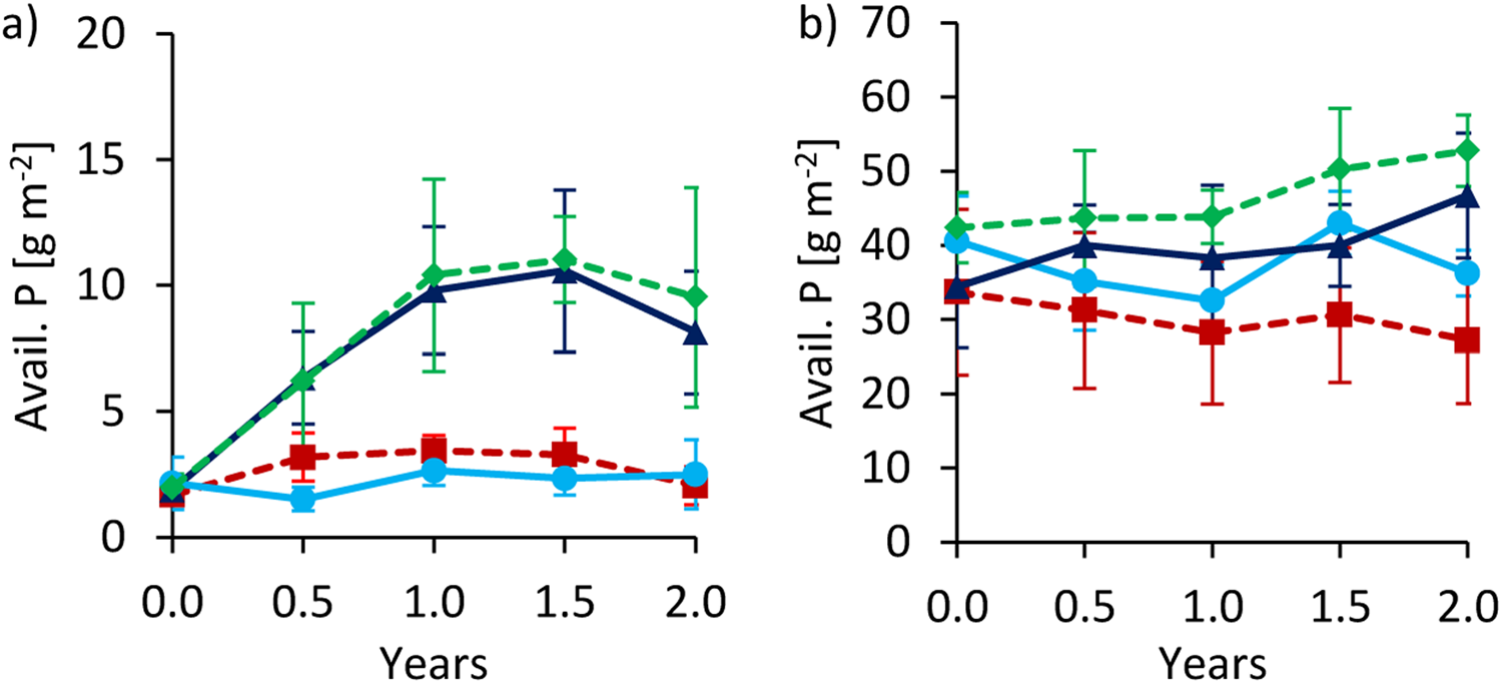
Changes of available P (Bray) over time at 0–20 cm depth under full irrigation for Tamale (**a**) and Ouagadougou (**b**). Means were calculated irrespective of irrigation water quality levels because they had no significant effects on available P (means ± sd; n = 8).

In Tamale, available P stocks increased with fertilization more than four-fold (Fig. 7), while biochar and water quality had no significant effects on available P over time. In line, total P stocks increased at a similar magnitude with fertilization (data not shown). Phosphorus additions by biochar (1.72 g m^−2^) were similar to initial available P stocks (1.93 ± 0.60 g m^−2^).

In Ouagadougou, available P (Fig. 7) remained largely unchanged over time across all treatments. Biochar P addition was negligible (2.8 g m^−2^) compared to available P stocks (37.7 ± 8.2 g m^−2^). Repeated fertilization with manure added 53.1 g P m^−2^ within two years, but the increase of available P measured in the same period was only marginal with 6.5 g m^−2^ on FP plots.

### CEC, exchangeable cations and ESP

**Figure 8:**
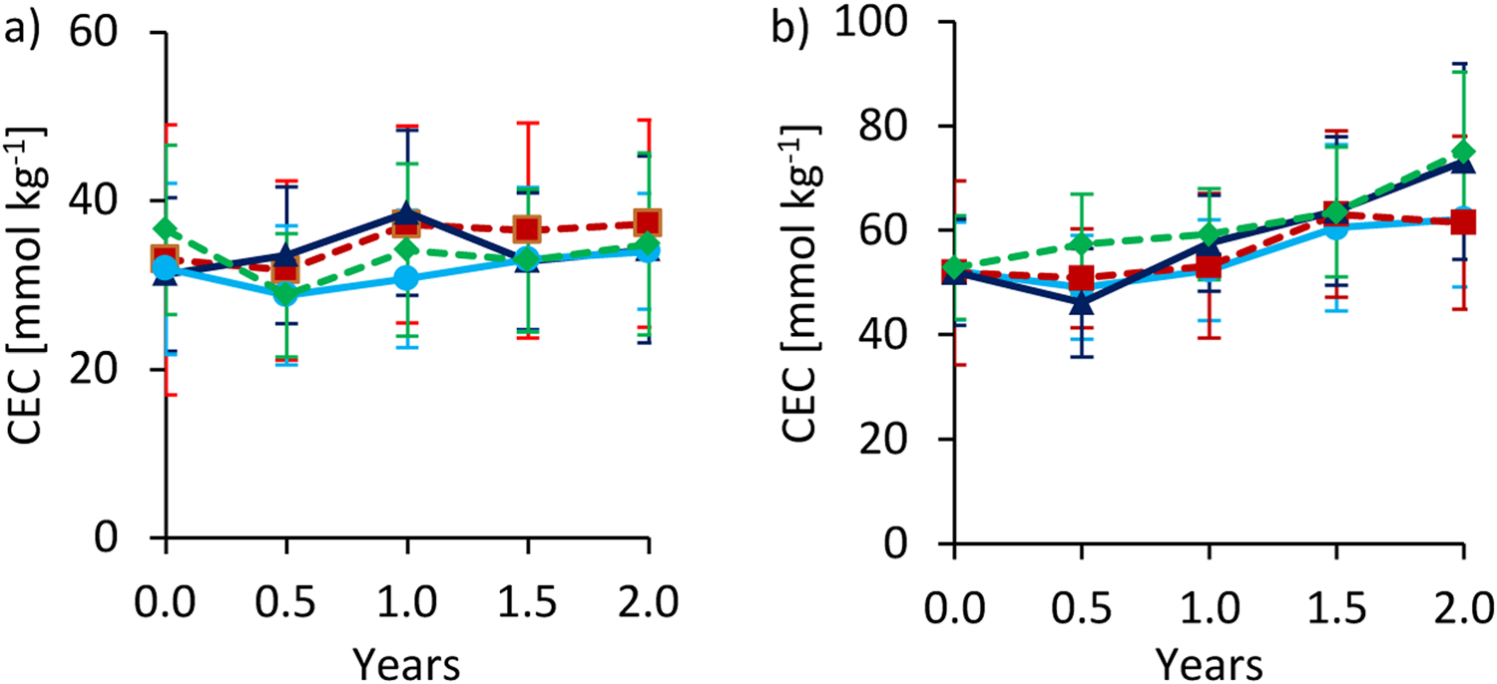
Changes of effective cation exchange capacity (CEC) over time at 0–20 cm depth under full irrigation for Tamale (**a**) and Ouagadougou (**b**). Means were calculated irrespective of irrigation water quality levels because they had no significant effects on CEC (means ± sd; n = 8).

In Tamale, effective CEC (Fig. 8), K^+^, Mg^2+^ and Ca^2+^ remained largely at the same levels over time for all treatments (data for cations not shown). In Ouagadougou, effective CEC slightly increased over time for all treatments, but the highest CEC increase was observed with fertilization (20 ± 7% higher than unfertilized treatments after two years). Among the cations, Mg^2+^ had the largest contribution to the increase of effective CEC with fertilization while Ca^2+^ had the largest contribution to the slight overall increase of effective CEC.

**Figure 9:**
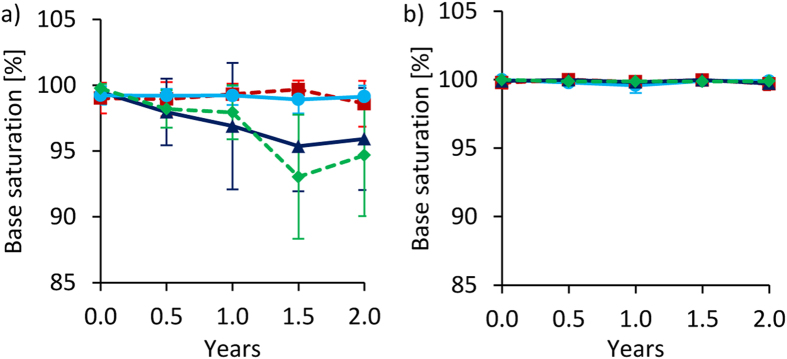
Changes of effective base saturation (BS) over time at 0–20 cm depth under full irrigation for Tamale (**a**) and Ouagadougou (**b**). Means were calculated irrespective of irrigation water quality levels because they had no significant effects on BS (means ± sd; n = 8).

In Tamale, fertilization (FP and FP+BC) led to a decline of effective BS from almost 100% down to 94.7 ± 1.3% (Fig. 9) and a corresponding increase of exchangeable Al^3+^ (data not shown) over time. In Ouagadougou, BS remained unchanged at 100% over time across all treatments (Fig. 9).

**Figure 10:**
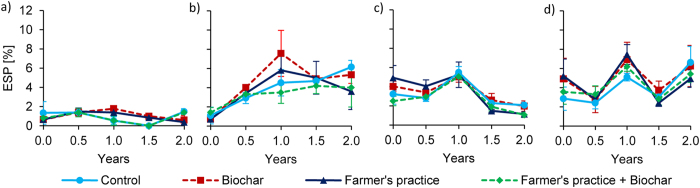
Changes of exchangeable sodium percentage (ESP) over time at 0–20 cm depth under full irrigation with clean water (**a**) and waste water (**b**) for Tamale as well as clean water (**c**) and waste water (**d**) for Ouagadougou (means ± sd; n = 4).

Waste water irrigation increased ESP almost five-fold in Tamale and 1.4 times in Ouagadougou after two years (Fig. 10). In Tamale, clean water irrigation had no influence on ESP. In contrast, in Ouagadougou, clean water irrigation decreased ESP after the change from well to tap water. A pronounced seasonal fluctuation of ESP with higher ESP after the dry season and lower ESP after the rainy season was observed in Ouagadougou.

## Discussion

### Effects of land use change on SOC and N dynamics

The net SOC decline of the control plots in Tamale (25% loss) was attributed to the land use change from previous rainfed to irrigated crop cultivation with increased soil moisture contents. The sensitivity of SOC decomposition rates to soil moisture contents is well known^15,16^. The impact of irrigation on SOC dynamics is particularly pronounced in soils with a low water holding capacity as given in the clay and SOC poor soils of the experimental sites. The lack of irrigation water quantity effects on SOC stocks suggested that even reduced irrigation added sufficient water to support microbial activity. Frequent tillage and the lack of organic fertilization further explain the observed SOC loss in Tamale. Irrespective of treatments, all plots were subject to this land use change-induced SOC decline.

The contrasting situation in Ouagadougou, where SOC and N stocks slightly increased over time in control plots, is also attributed to land use change. Before the onset of the experiment the field had been subject to carrot cultivation and motor pump irrigation with excessive water quantities. Three land use change aspects likely increased biomass inputs in the soil and decreased soil moisture contents and, thus, offer explanations for the net SOC and N increase: First, biomass input in soil is lower for carrots than for other vegetables because not only shoots but the complete plant is harvested. Second, the experimental area was not fenced before, thus, small ruminants (goat and sheep) likely have entered the site and have eaten harvest residues. Last, the change from motor pump to hand held watering can irrigation likely led to lower, plant oriented irrigation quantities and, thus, lower soil moisture contents.

The slight increases of exchangeable Ca^2+^ and effective CEC over time across all treatments in Ouagadougou are attributed to the slight increase of SOC which provides reactive surfaces. Last, increasing Ca^2+^ stocks induced the slight pH increase which was observed over time across all treatments.

### Effects of biochar on SOC dynamics

Remarkably large net SOC losses were observed in biochar amended plots at the first re-sampling campaign after half a year at both sites (35% in Ouagadougou and 27% in Tamale). Further, in Tamale, a continuous net SOC loss of biochar plots was determined throughout the measurement period (46% loss after 2 years). However, assuming that BC plots in Tamale were subject to the same land use change induced decline of native SOC as control plots, SOC loss of BC plots narrowed down to 16% after 0.5 years and 35% after two years.

Potential SOC loss pathways at 0–20 cm depth are decomposition, leaching, erosion and bioturbation^17^. The fast initial decline of the C/N ratios of BC and FP+BC plots after the addition of the C rich and N poor biochars suggest that a large proportion of C loss derived from biochar-C. The extrapolation of lab experiments on C decomposition and leaching (data not shown) provided further evidence on the magnitude of these two pathways.

The column experiment demonstrated higher DOC leaching under soils amended with biochar than in the control soil. In addition, excessively high leaching of fine particulate (particle size powder-like >0.45 µm) and dissolved organic C was observed in biochar amended soil during the initial irrigation events in the column experiment (J. Werner, unpublished). Especially corn cob biochar had a high proportion of fine sized particles after crushing to <2 mm. These particles likely made corn cob biochar more prone to leaching which may explain the higher initial SOC losses of corn cob biochar than of rice husk biochar.

The observed high initial rates suggested that leaching of particulate biochar C is a rapid process which ceased within days or weeks in the field. The high sand and low SOC contents at both sites favored high leaching rates. Lacking differences in SOC contents over time in the well drained 20–40 cm subsoil suggested that accumulation of relocated biochar particles occurred below the sampled depth. These results indicated that leaching contributed to SOC loss after biochar addition whereby this effect was rather short- than long-term. In contrast to our laboratory findings, in other studies leaching of biochar as particulate or dissolved C was found to be small (1% of total biochar-C^18^) or not detectable^19^.

CO_2_ evolution increased with increasing biochar addition rates. These results support that decomposition contributed to the rapid SOC loss after biochar addition. Despite its generally recalcitrant nature, biochar has a labile C fraction which is subject to decomposition within months to years^20^. In other studies, biochar losses by decomposition accounted for only 2.2% after two years in a field trial in Colombia^18^ and 0.5% per year in a laboratory trial^19^. However, the biochar types used in our study had very high and similar contents of volatile matter, indicating large labile C fractions (23% for rice husk biochar, 20% for corn cob char; Table 2). Further, with high temperatures and moderate moisture contents throughout the year, low clay contents, high aeration and a slightly acid pH, conditions for microorganisms and C decomposition were favorable at both sites. In addition, biochar may have led to an increased decomposition of native SOC by priming^18,20^ which then contributed to gross C decomposition. In Tamale, fully irrigated BC plots had 1.1 ± 0.4 times higher and reduced irrigated BC plots had 1.5 ± 1.5 times higher soil moisture contents than control plots in the dry season (E. K. Akoto-Danso, unpublished). Given the moisture sensitivity of SOC decomposition, these differences support higher decomposition rates in biochar amended soil compared to control plots.

**Table 2:** Elemental contents of biochar and organic manure.

	Total C	Total N	CN	P	K^+^	Ca^2+^	Mg^2+^	Na^+^	pH (CaCl_2_)	Ash	Volatile matter
	g kg^−1^	g kg^−1^		g kg^−1^	g kg^−1^	g kg^−1^	g kg^−1^	g kg^−1^		%	%
Rice husk biochar	424.0	6.0	70.7	0.86	0.98	1.57	0.95	0.28	9.1	45.2	23.2
Corn cob biochar	683.6	8.8	77.7	1.41	3.30	3.51	1.15	0.43	10.3	18.5	19.6
Cattle manure^1^	166.9	13.1	12.8	3.75	6.53	7.84	8.93	nd	nd	nd	nd
	*54*.*9*	*3*.*4*		*0*.*78*	*2*.*16*	*1*.*47*	*1*.*33*				

^1^Means ± SD (in italics) over all crops which received manure (n = 11).

^2^Biochar was applied in a single initial addition; Manure was applied for each crop (except one) over two years.

Soil erosion is another potential pathway for biochar and native SOC loss^18^. Despite the plane topography of our experimental area, occasionally heavy rain events led to sealing of the silty soil surface and transport of biochar particles to depressions within the plots. The high susceptibility of charred material to erosion due to its light weight has been described for other sites^21^. However, frequent loosening of the soil surface and tillage occurred as part of the intensive cultivation regime throughout the experimental time, supporting a homogeneous infiltration within the plots. The low impact of soil erosion on redistribution of biochar particles was supported by two findings: First, given the flat terrain, not only biochar depleted but also biochar enriched plots would have been sampled if severe relocation of biochar would have taken place. However, the lack of large differences of standard deviations of mean SOC stocks of biochar plots indicated that biochar relocation by erosion was small. Second, the large differences of C/N ratios between control and BC plots beyond 0.5 years suggest that native SOC loss was equal or even higher than biochar-C loss.

Another pathway for SOC loss from the top 20 cm may be bioturbation, induced by abundant termite populations. However, for BC plots, the similar subsoil SOC stocks over time at both sites indicated that relocation of biochar from the top into the subsoil was negligible.

Hence, we conclude that the rapid initial C loss was dominated by leaching and decomposition of biochar-C fractions while the longer term SOC loss in biochar plots in Tamale was likely dominated by decomposition of native SOC and biochar-C. Other biochar properties such as pH and C/N ratio and site characteristics were similar between the two biochar types and the two sites. High initial biochar-C losses may be avoided if biochar is not added in one single application but repeatedly, such as after each harvest, at lower doses to allow the slow paced interactions with soil minerals, aggregates and soil fauna to take place^22^. At the same time, small but repeated biochar application rates are also more practical for the majority of smallholders who face limitations in biomass availability.

### Effects of organic fertilization on SOC and N dynamics

In Ouagadougou, the continuous application of manure led to an increase of SOC stocks beyond those of BC plots. Interestingly, half a year after the initial application, net SOC losses were higher on corn cob BC plots (35%) than on FP plots with manure addition (11%) on a calculation basis (biochar or manure plus SOC). Apparently, the biochar used in our study had a larger labile C fraction which was more susceptible to rapid losses by leaching and decomposition than manure-C. However, in the longer term (0.5 to 2.0 years) BC plots had no further net SOC loss in Ouagadougou while SOC loss in manure plots remained at similar rates (18%) as in the first half year. This is in line with other reports, since manure-C usually has a faster turnover rate than biochar-C^11^. However, manure-C losses vary highly depending on the manure composition, application on top or mixing in the soil as well as microbial and faunal activity^23^. Low manure-C losses in the present study were attributed to the high stability of the cattle manure-C. Similarly low manure-C losses (24–31% of applied manure-C) were observed by Markewich *et al*.^24^ in Kenya in a litterbag experiment.

Similar rates of C mineralization for control and the manure treatment were supported by equally low C/N ratios. With C/N ratios of around 10, conditions were favorable for SOC decomposition. Over time, manure narrowed down the C/N ratio of the FP+BC plots to the level of FP and control plots because of mixing C rich biochar with N rich manure. In contrast to C and in line with declining C/N ratios, no net N loss was observed when comparing manure-N inputs with N stocks of FP plots over time. Apparently, plant N needs were satisfied by the inorganic N fertilizer, which was added in combination with manure.

Thus, to maintain high SOC stocks, manure should be regularly added, while in the present study N accumulated with repeated manure addition. With low C and N, CEC and high acidification rates manuring is highly recommended for Tamale.

### Effects of biochar on N dynamics

In Tamale, BC plots had 18% higher N stocks than control plots, without N inputs by fertilizer and waste water. These higher N stocks were partially attributed to biochar-N additions (12% relative to initial N stocks). Thus, further mechanisms favored N retention in biochar amended plots, such as increased NH_4_ sorption, decreased NO_3_ leaching or lower plant N uptake but further studies are necessary to analyze the involved mechanisms. Nitrogen retention by biochar was also reported by Steiner *et al*.^25^ and Biederman and Harpole^12^ and is in line with the theoretical explanations by Clough and Condron^26^.

In contrast to rice husk biochar addition, corn cob biochar had no net effects on N stocks, despite proportionally similar N additions to soil as rice husk biochar in Tamale. Low N sorption capacity of corn cob biochar may explain this pattern but further studies are necessary to understand sorption and desorption mechanisms and gross fluxes of N fractions between soils, plants and atmosphere.

### Effects of inorganic N fertilization and irrigation on SOC and N dynamics

The cumulative inorganic N inputs (NO_3_ and NH_4_) by waste water and fertilization over two years were of similar magnitude as the total N supplies of the control treatments at both sites. Despite these high inputs and corresponding biomass returns soil N stocks did not increase, while in Tamale total N stocks even decreased over time across all treatments. Apparently, N losses by leaching, plant uptake and volatilization were as large as or even larger than inorganic N inputs. The lack of a waste water effect on soil N was in line with Heidarpour *et al*.^27^ and Pinto *et al*.^28^.

Further, no net effect of waste water on SOC stocks was detected. Other studies found inconsistent effects on SOC, both an increase^29–31^ or a decrease^32^ depending on the magnitude of direct C addition and indirect effects of waste water on C mineralization.

### Effects of fertilization and biochar additions on further soil properties

The strong acidification under fertilization (FP and FP+BC) in Tamale was attributed to nitrification of NH_4_ inputs from the applied inorganic fertilizers. With low contents of clay and SOC, soil buffer capacity was low at both experimental sites. The decline of pH went along with an increase in exchangeable Al^3+^ which led to a decline of effective BS. Acidification usually releases Al^3+^ from clay minerals^33^. The pH did not decrease below 4.2 after 1.5 years likely because functional groups of Al^3+^ oxides served as buffer which is a common soil reaction mechanism at this pH level.

In Ouagadougou, slightly lower pH values for fertilized than unfertilized treatments were attributed to nitrification of the inorganic component of the fertilizer mix, too. The decline of pH was not as strong as in Tamale, despite twice as much inorganic fertilizer-N inputs in Ouagadougou, because organic fertilization likely increased the buffer capacity.

Both biochars had no net liming effect in the present study, despite their high ash contents. Likely, ash derived basic cations were washed out quickly and thus, were not captured by the bi-annual measurement intervals. This is in contrast to other studies, where biochar caused a pH increase due to addition of ash rich in basic cations^12,13^. Gaskin *et al*.^34^ reported a liming effect in the first year after biochar application and a subsequent decline.

In Tamale, available P increased under fertilization at similar quantities as total P. Apparently, P which was applied in excess of plant needs was retained in the P depleted soils. No effect of acidification on changes in P availability was observed. In Ouagadougou, available P increased slightly, despite high P additions by manure. The gap between added and measured available P (46.6 g m^−2^) most likely reflected plant uptake of manure-P. Biochar had no effect on P availability at both sites despite P additions with biochar. This is in contrast to previous studies which reported a P increase after biochar addition^12,35^. Fertilization with P was less relevant in Ouagadougou than in Tamale due to the high available P supplies of soils in Ouagadougou.

The slightly increased effective CEC under manure fertilization was attributed to Mg^2+^ inputs by manure and increasing SOC stocks over time. Effective CEC of the two biochars were an order of magnitude higher than the CEC of the respective soils but the application rate was too small to cause a significant impact on soil CEC. Most studies report an increase of CEC after char addition^11,13^. A more pronounced effect of biochar on CEC may develop in soils over time. Several studies indicate that CEC may increase by microbial and abiotic oxidation of biochars^22,36^.

### Effects of irrigation on pH and cations

The seasonal ESP fluctuations in Ouagadougou were explained by the seasonality of Na^+^ rich waste water irrigation and the dilution of waste water with rainwater in the rainy season. Thus, during the rainy seasons a net decline of ESP occurred while during the dry season a net increase of ESP was observed.

In Tamale, the small pH increase under waste water irrigation was explained by the high Na^+^ and associated OH^-^ inputs. In Ouagadougou, the pronounced effect of water quality on pH after two years was also a consequence of waste water Na^+^ inputs. Before the change of the clean water source from well to tap water after the first year (and the subsequent rainy season), no pronounced waste water effects occurred, likely because the differences in properties of well and waste water were small. Increasing Na^+^ stocks under waste water irrigation are commonly reported^9,30,32^. In extreme cases, irrigation leads to salinization as found at adjacent sites in Ouagadougou^9^. Other studies on the effects of waste water irrigation on pH found both, increases^28,37^ and decreases^30,32^ or no effects^27,29^ on pH, depending on the initial soil pH and the ratio of inputs and losses of basic cations.

The lack of waste water irrigation effects on available and total P was in line with Heidarpur *et al*.^27^, Lado and Ben-Hur^10^ and Aydin *et al*.^29^. In contrast, Kiziloglul *et al*.^38^ observed an increase in P in a chronosequence study over 33 years. The lack of a waste water effect on cations and base saturation was also in line with Heidarpur *et al*.^27^ and Bedbabis *et al*.^30^. In literature over all, conclusions for waste water effects on exchangeable cations and available P are inconsistent. Mostly, waste water effects are attributed to the ratio between inputs, initial or control soil properties and elemental losses by leaching and crop uptake.

## Methods

### Study areas

The Tamale field experiment is located at 9°28′29N, 0°50′53W and the Ouagadougou field experiment at 12°24′16N, 1°28′40W. At both locations the climate is semi-arid with average annual rainfalls of 1111 mm in Tamale and 788 mm in Ouagadougou and similar mean annual temperatures of 27.9 and 28.2 °C, respectively^39^. The rainy season is longer in Tamale (April to October) than in Ouagadougou (May to September). While the topography of both experimental sites is plain, the site in Ouagadougou is situated on an elevated plateau in a floodplain. Both sites are outside of flooding zones and groundwater fluctuations. Before the onset of the experiments soil properties within the experimental sites and the wider surroundings were mapped in order to identify sites with similar soil texture, structure and color and a similar minimum soil depth of 40 cm. Soil types were a Petroplinthic Cambisol in Tamale and a Haplic Lixisol (Cutanic) in Ouagadougou according to the WRB^40^ classification. Initially, both sites were poor in SOC (4.1 and 5.7 g kg^−1^ in Tamale and Ouagadougou, respectively), total N (0.4 and 0.6 g kg^−1^), effective CEC (33 and 52 mmol_c_ kg^−1^) and clay contents (5% and 8%). The site in Tamale is underlain by a petroplinthic hard pan whereas the site in Ouagadougou is derived from floodplain sediments. Prior to setting up the experiments, the site in Ouagadougou was used for intensive vegetable cultivation while the site in Tamale had been used for rainfed maize production. Oral records indicated that long-term previous land use was identical within the experimental areas and each area belonged to a single owner.

### Experimental setup

Local farmer’s usual vegetable farming practices (FP) are characterized by crop specific organic and inorganic fertilization and irrigation with untreated waste water. At the two sites, two fertilization levels (fertilization according to FP and an unfertilized control) and two biochar levels (0 and 2 kg m^−2^ biochar addition) were irrigated with two water quality levels (untreated waste water and clean water) and two water quantity levels (“full”, that is the typical irrigation quantity and “reduced”, that is 2/3 of the full amount) in full factorial split-plot designs with four blocked replicates (a total of 64 plots per site). The randomization of the treatment allocation to plots occurred in two steps. First, each block was divided into four main-plots to which the four water levels were randomly allocated. Second, main plots were split into four sub-plots to randomly accommodate the four fertilization and biochar levels. Sub-plot size was 8 m² (2 × 4 m).

Biochar was produced by slow pyrolysis at around 500 °C in a local kiln. Raw materials were rice husks in Tamale and corn cobs in Ouagadougou. These raw materials are nutrient poor, dry and have a low decomposition rate which may lead to decreasing nitrogen availability for plants after field application. Both feedstocks were abundantly available with only few alternative uses. Biochar was incorporated at 0–20 cm depth at a quantity of 2 kg m^−2^ at both sites which was an addition of 0.71% w/w rice husk biochar and an addition of 0.95% w/w corn cob biochar relative to soil mass (Table 2). While the particle size of rice husk biochar was small enough for direct field application, corn cob biochar was crushed to <2 mm prior to use.

In Tamale, waste water came from a large military barrack while clean water came from a tap for potable water. In Ouagadougou, the origin of waste water was the large open channel which passes the metropolis from West to East. Clean water came from a well and from February 2015 on from a tap, due to water shortage in the well. At both sites, concentrations of all nutrients were higher in waste water compared to clean water and waste water was more nutrient rich in Tamale than in Ouagadougou (Table 3). The largest differences were found for N and P in Tamale, with 80 times higher N and 175 times higher P concentrations in waste compared to clean water. Sodium had 10 and 7 times higher concentrations in waste water compared to clean water in Tamale and Ouagadougou, respectively. Both field experiments were irrigated by hand with watering cans. Over two years, total water quantity under full irrigation was 1915 l m^−2^ in Tamale and 3005 l m^−2^ in Ouagadougou.

**Table 3:** Elemental concentrations of waste and clean water in Tamale and Ouagadougou. Means over all crops ± SD (in italics).

	**N** ^1^	**PO**_**4**_ **-P**	**Ca** ^**2+**^	**Mg** ^**2+**^	**K** ^**+**^	**Na** ^**+**^	**pH**
	mg l^−1^	mg l^−1^	mg l^−1^	mg l^−1^	mg l^−1^	mg l^−1^	
**Tamale**							
Clean water	0.4	0.1	9.4	2.3	1.7	2.0	7.4
	*0*.*2*	*0*.*0*	*1*.*9*	*0*.*6*	*0*.*9*	*0*.*8*	*0*.*7*
Waste water	31.9	8.9	28.2	6.0	8.4	19.2	7.6
	*14*.*6*	*6*.*3*	*5*.*8*	*0*.*6*	*5*.*7*	*3*.*9*	*0*.*7*
**Ouagadougou**							
Clean water	0.8	0.2	15.7	3.7	8.2	6.0	8.3
	*0*.*8*	*0*.*0*	*9*.*1*	*1*.*6*	*2*.*9*	*2*.*1*	*0*.*5*
Waste water	3.9	0.8	22.5	4.8	44.0	38.8	8.4
	*1*.*5*	*0*.*3*	*4*.*5*	*1*.*8*	*29*.*7*	*17*.*7*	*0*.*6*

^1^N = NO_3_-N + NH_4_-N.

Vegetable types and cultivation times were synchronized between Tamale and Ouagadougou, except for two additional crops which were cultivated in Tamale (*Zea mays* and *Corchorus*) while the plots in Ouagadougou were left fallow. The planted vegetables were *Lactuca sativa* L. (3 times), *Amaranthus cruentus* (3 times), *Corchorus olitorius* L. (twice), *Brassica oleracea* L. (once), *Hibiscus sabdariffa* L. (once) and *Daucus carota* L. (once).

Types and quantities of fertilizers were applied according to local farmers’ practice. In Tamale, fertilization was purely inorganic with NPK 15-15-15 (200 to 563 kg ha^−1^ per crop) for all crops except for two crops (*Corchorus*) where urea was applied at 247 and 256 kg ha^−1^ instead of NPK. In Ouagadougou, a combination of urea (70 to 375 kg ha^−1^ per crop) and cattle manure (9 to 20 t ha^−1^ per crop) was applied to all crops apart from *Hibiscus sabdariffa* L. which received only urea. Over two years, fertilization of inorganic N was twice as much in Ouagadougou (1053 kg N ha^−1^) than in Tamale (586 kg N ha^−1^).

Nursery of seedlings, planting distance, weeding and further crop management was done according to local farmers’ practice for all plots. Tillage was done manually to 20 cm soil depth.

The presented data belong to a series of forthcoming publications which cover plant growth and soil matter dynamics, including crop uptake, leaching and gaseous emissions of elements from the same field experiments.

### Soil sampling and analyses

Soil samples were collected bi-annually after each rainy and dry season at 0–20 cm depth for two years. Soils at 20–40 cm depth were sampled initially and after two years. The first sampling occurred at the end of the dry season 2013/14, after plot demarcation and prior to treatment installation. For each plot a composite sample, consisting of samples from two locations within the plot, was taken for analysis. Soil samples were dried, disaggregated and passed through a 2 mm sieve prior to analysis for total C and N by dry combustion (Vario EL Elementar Analysesysteme GmbH, Hanau, Germany) and pH in CaCl_2_ solution (1:2.5 w/v). For treatments under full irrigation, available P (Bray), exchangeable Ca^2+^, Mg^2+^, K^+^, Na^+^ and Al^3+^ (by NH_4_Cl extraction), total P and heavy metals (by HNO_3_ extraction) were measured by ICP-OES (Ciros CCD, Spectro Analytical Instruments GmbH, Kleve, Germany). Effective CEC (mmol_c_ kg^−1^) was calculated as sum of the exchangeable cations, accounting for their valence. Exchangeable sodium percentage (*ESP;* %) was calculated according to equation 1.

1 $$ESP=\frac{Na}{CEC}\cdot 100$$

Effective base saturation (*BS*; %) was calculated according to equation 2, accounting for the valence of exchangeable cations.

2 $$BS=\frac{(Ca+Mg+K+Na)}{CEC}\cdot 100$$

Bulk density was measured initially in triplicates at one point in the center of each block and plot-specific in triplicates after two years. Bulk density was not measured more frequently to avoid disturbance of the plots. At both locations, soils contained plinthite nodules with a diameter of >2 mm. The volume-% of the fraction >2 mm (stone content) was measured during sample preparation by accounting for the weights of fragments >2 mm, fine soil <2 mm, soil bulk density and the density of the fragments >2 mm (2.65 g cm^−3^). All results were reported for oven dry (105 °C) soil. SOC and nutrients were reported as stocks (g or kg m^−2^) by multiplication of SOC and nutrient contents with bulk density and soil depth after subtraction of the stone contents from the soil mass.

### Statistics

Normal distribution of residuals was tested with the Kolmogorov-Smirnov test and visually assessed with qq-plots. Where necessary, data were log transformed prior to statistical analysis. To account for the multi-factorial design, mixed model analyses (proc mixed) with main factors and interactions between them were conducted with a main-plot and a sub-plot error as random effects. The main plot error was represented by water quality*water quantity*block and accounts for the restricted randomization in the split-plot design. An autoregressive AR(1) model was used to account for correlations within the time series of measurements taken from the same plots. Significances (p ≤ 0.05) of differences between treatments were determined by comparing least square means adjusted by the Tukey-Kramer method. Data are reported as means ± standard deviation. Statistics were conducted with SAS (SAS Institute Inc., Carey, NC, USA) and figures were produced with Microsoft Excel 2010.

### Permisson for unpublished personal communications

Permission for the citation as personal communication was given by all data producers: E. K. Akoto-Danso is member of the author team and written permissions from A. Neuser and J. Werner are attached. A. Neuser and J. Werner were involved in the experiments as M.Sc. students and were supervised by the author team.
