# Author Correction: Effects of biochar, waste water irrigation and fertilization on soil properties in West African urban agriculture

**DOI:** 10.1038/s41598-018-22637-7

**Published:** 2018-03-08

**Authors:** Volker Häring, Delphine Manka’abusi, Edmund K. Akoto-Danso, Steffen Werner, Kofi Atiah, Christoph Steiner, Désiré J. P. Lompo, Samuel Adiku, Andreas Buerkert, Bernd Marschner

**Affiliations:** 1 Institute of Geography, Department for Soil Science and Soil Ecology, Ruhr-Universität Bochum, Universitätsstr. 150, D-44780 Bochum, Germany; 2 Organic Plant Production and Agroecosystems Research in the Tropics and Subtropics, Universität Kassel, Steinstr. 19, D-37213 Witzenhausen, Germany; 3 Department of Soil Science, University of Ghana, Accra, Ghana

Correction to: *Scientific Reports* 10.1038/s41598-017-10718-y, published online 06 September 2017

This Article contains errors in Figures 1, 4, 5, 7, 8 and 9, where the keys were omitted. The correct Figures 1, 4, 5, 7, 8 and 9 appear below as Figures 1, 2, 3, 4, 5 and 6 respectively.

**Figure 1:**
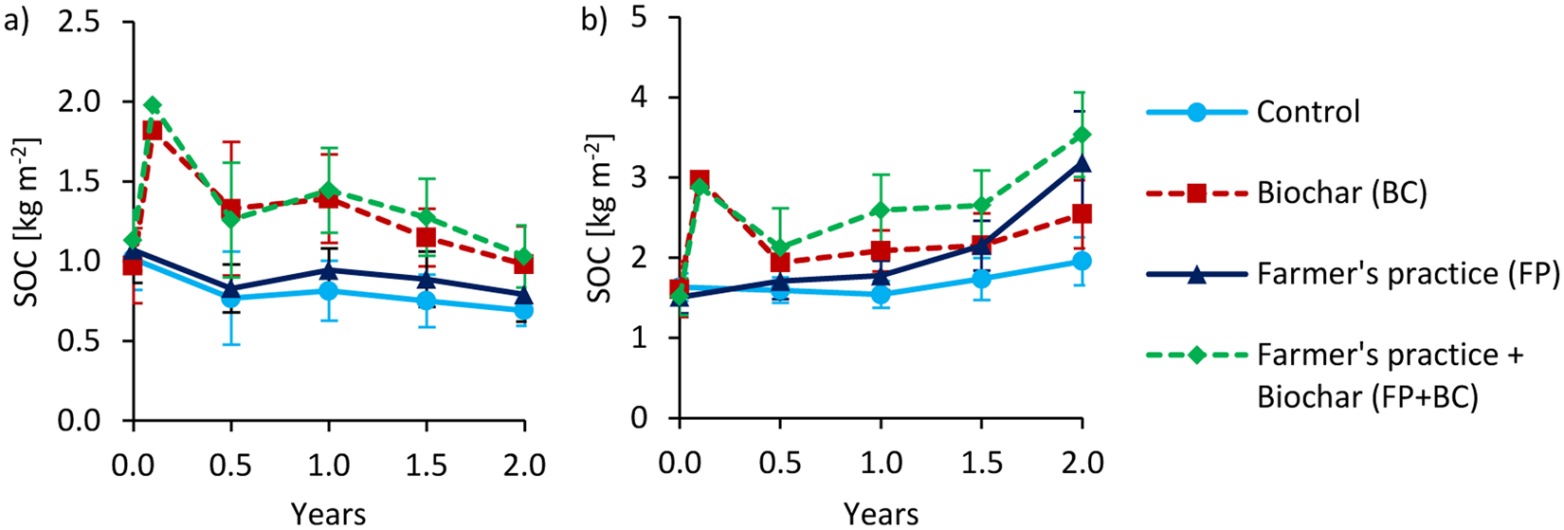
Changes of soil organic carbon stocks over time at 0–20 cm depth for Tamale (**a**) and Ouagadougou (**b**). Means were calculated irrespective of irrigation water quantity and quality levels because they had no significant effects on SOC stocks (means ± sd; n = 16). Values after biochar additions (between 0 and 0.5 years) are calculated and have no standard deviation.

**Figure 2:**
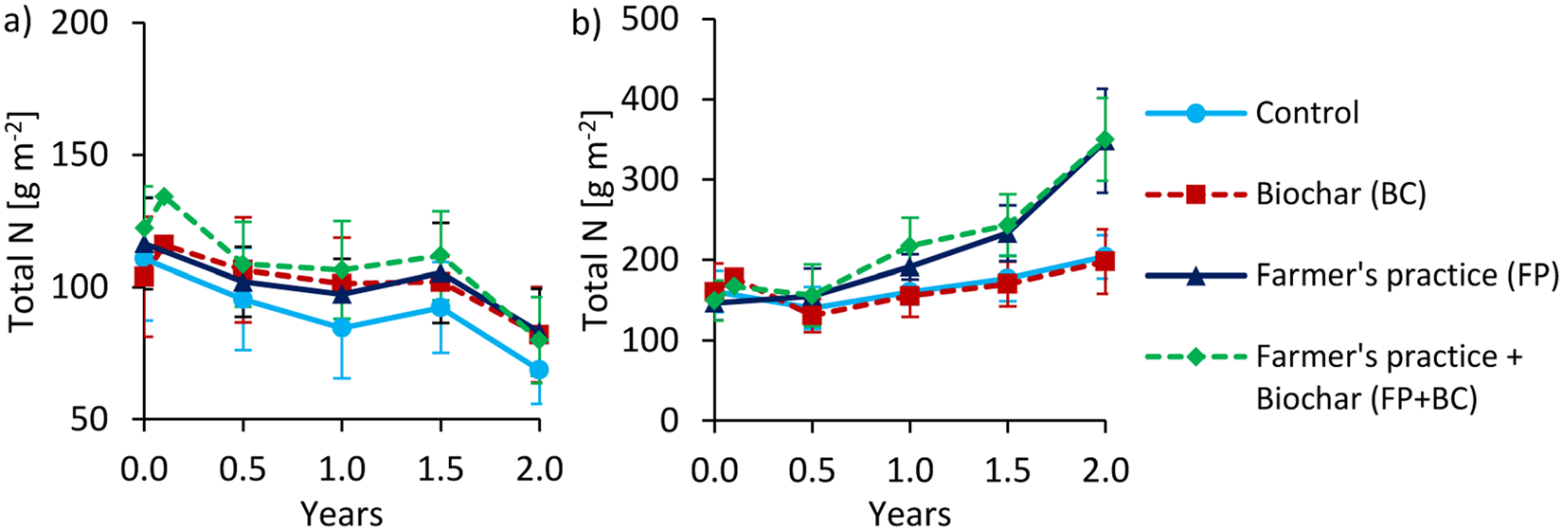
Changes of total N stocks over time at 0–20 cm depth for Tamale (**a**) and Ouagadougou (**b**). Means were calculated irrespective of irrigation water quantity and quality levels because they had no significant effects on N stocks (means ± sd; n = 16). Values after biochar additions (between 0 and 0.5 years) are calculated and have no standard deviation.

**Figure 3:**
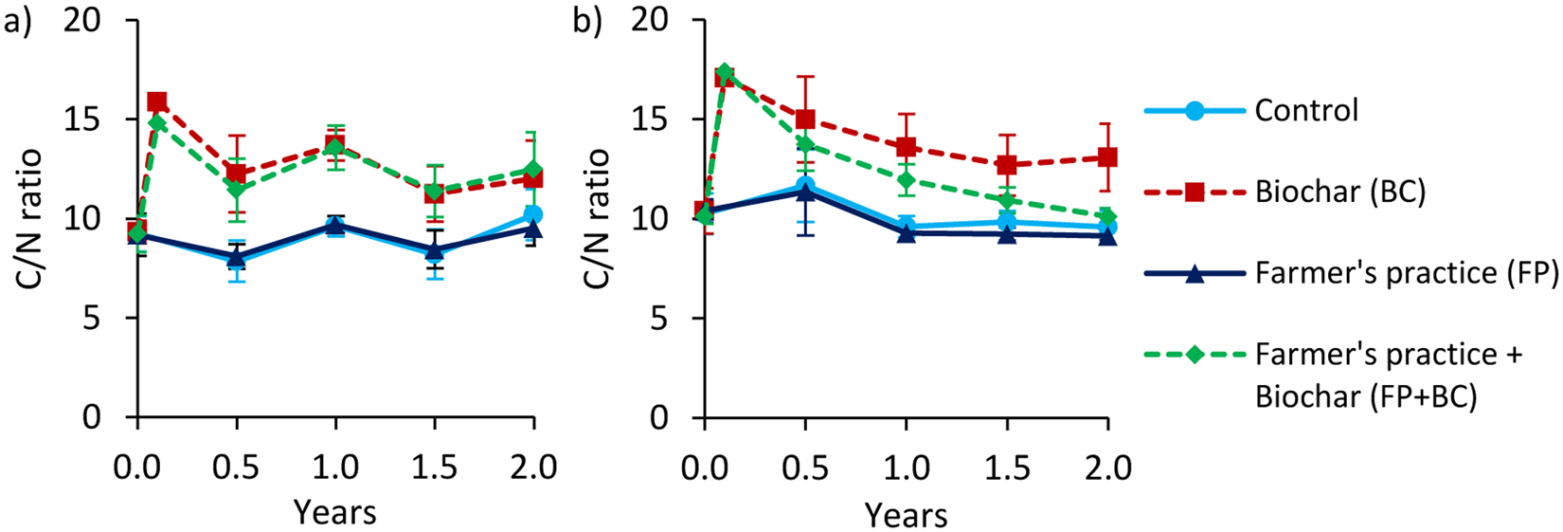
C/N ratio changes over time at 0–20 cm depth for Tamale (**a**) and Ouagadougou (**b**). Means were calculated irrespective of irrigation water quantity and quality levels because they had no significant effects on C/N ratios (means ± sd; n = 16). Values after biochar additions (between 0 and 0.5 years) are calculated and have no standard deviation.

**Figure 4:**
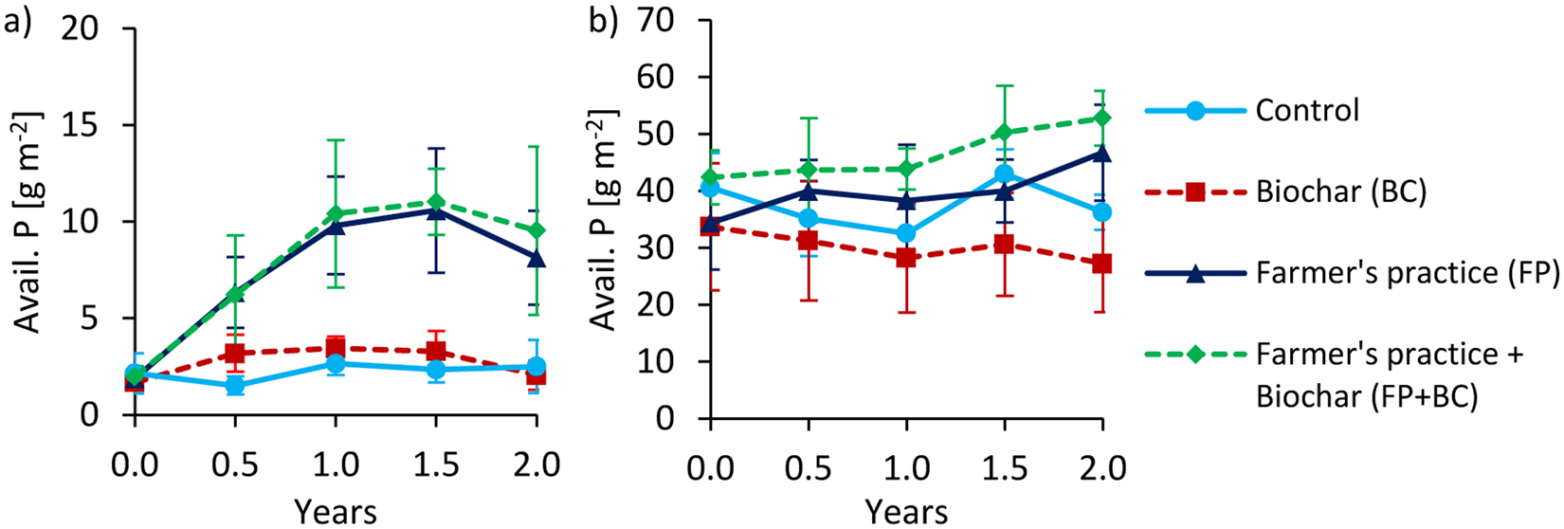
Changes of available P (Bray) over time at 0–20 cm depth under full irrigation for Tamale (**a**) and Ouagadougou (**b**). Means were calculated irrespective of irrigation water quality levels because they had no significant effects on available P (means ± sd; n = 8).

**Figure 5:**
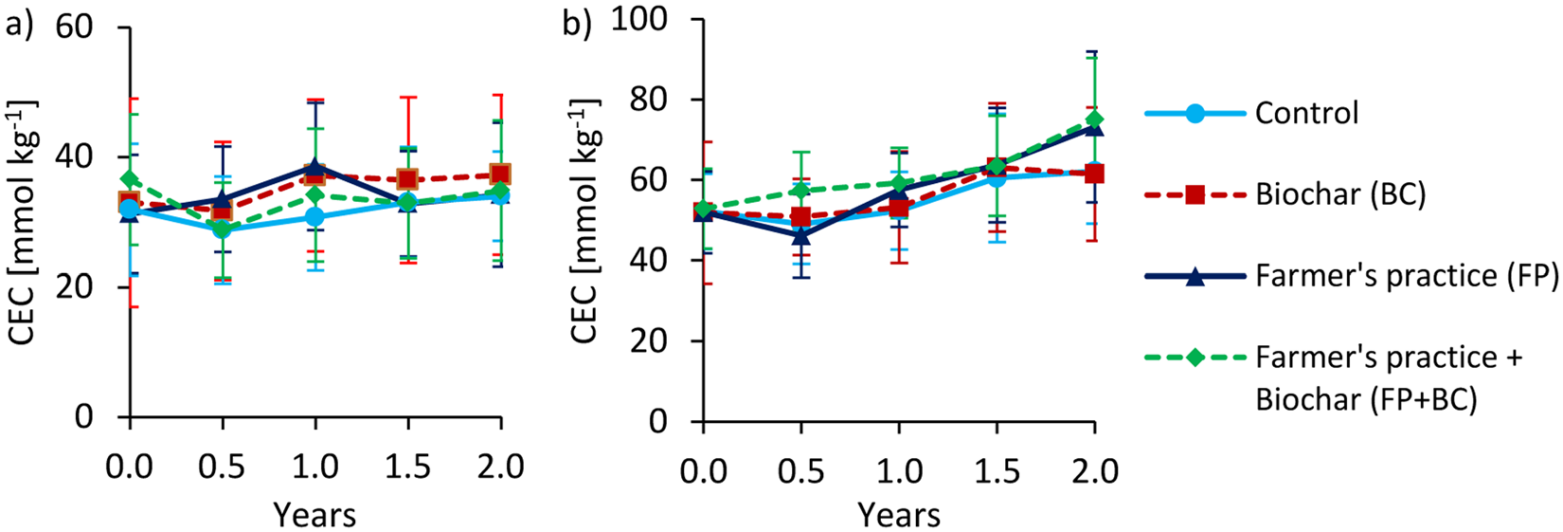
Changes of effective cation exchange capacity (CEC) over time at 0–20 cm depth under full irrigation for Tamale (**a**) and Ouagadougou (**b**). Means were calculated irrespective of irrigation water quality levels because they had no significant effects on CEC (means ± sd; n = 8).

**Figure 6:**
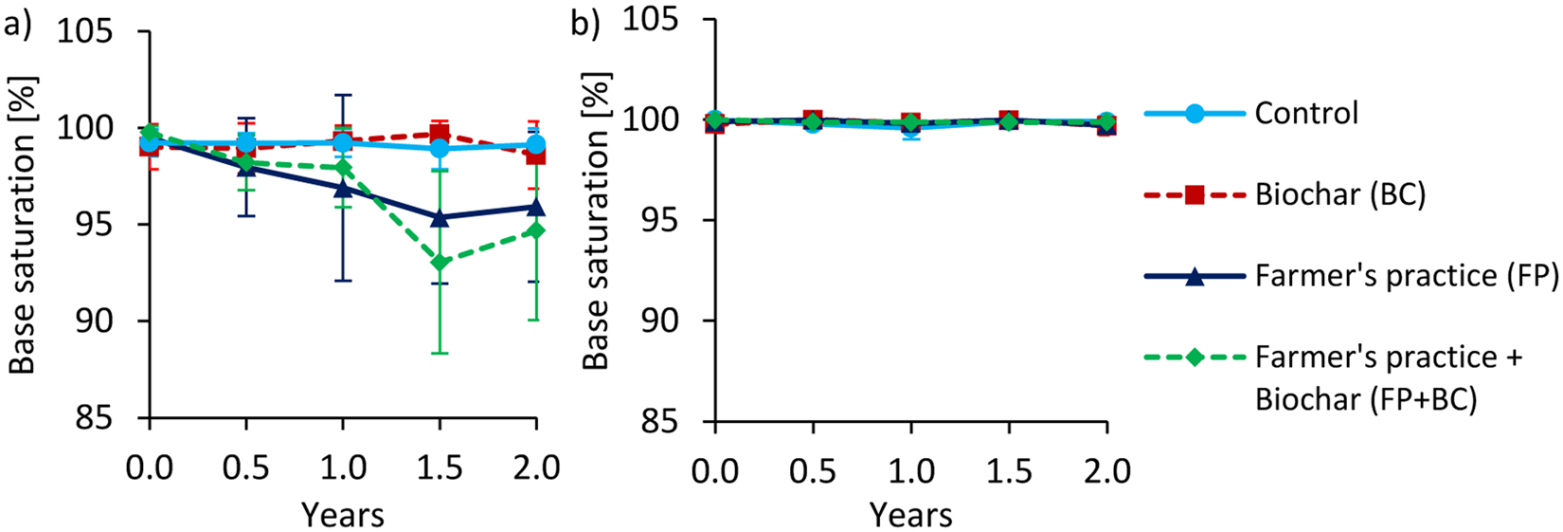
Changes of effective base saturation (BS) over time at 0–20 cm depth under full irrigation for Tamale (**a**) and Ouagadougou (**b**). Means were calculated irrespective of irrigation water quality levels because they had no significant effects on BS (means ± sd; n = 8).

